# Enhanced non-vitreous cryopreservation of immortalized and primary cells by ice-growth inhibiting polymers†
†Electronic supplementary information (ESI) available. See DOI: 10.1039/c6bm00129g



**DOI:** 10.1039/c6bm00129g

**Published:** 2016-05-06

**Authors:** Robert C. Deller, Jeffrey E. Pessin, Manu Vatish, Daniel A. Mitchell, Matthew I. Gibson

**Affiliations:** a Department of Chemistry , University of Warwick , CV4 7AL , UK; b Molecular Organisation & Assembly of Cells DTC , University of Warwick , CV4 7AL , UK; c Department of Medicine , Albert Einstein College of Medicine , Bronx , New York , USA; d Department of Molecular Pharmacology , Albert Einstein College of Medicine , Bronx , New York , USA; e Nuffield Department of Obstetrics & Gynaecology , University of Oxford , OX3 9DU , UK; f Warwick Medical School , University of Warwick , CV4 7AL , UK

## Abstract

Cell cryopreservation is an essential tool in modern biotechnology and medicine. The ability to freeze, store and distribute materials underpins basic cell biology and enables storage of donor cells needed for transplantation and regenerative medicine. However, many cell types do not survive freezing and the current state-of-the-art involves the addition of significant amounts of organic solvents as cryoprotectants, which themselves can be cytotoxic, or simply interfere with assays. A key cause of cell death in cryopreservation is ice recrystallization (growth), which primarily occurs during thawing. Here it is demonstrated that the addition of ice recrystalization inhibiting polymers to solutions containing low (non vitrifying) concentrations of DMSO enhance cell recovery rates by up to 75%. Cell functionality is also demonstrated using a placental cell line, and enhanced cryopreservation of primary rat hepatocytes is additionally shown. The crucial role of the polymers architecture (chain length) is shown, with shorter polymers being more effective than longer ones.

## Abstract 2

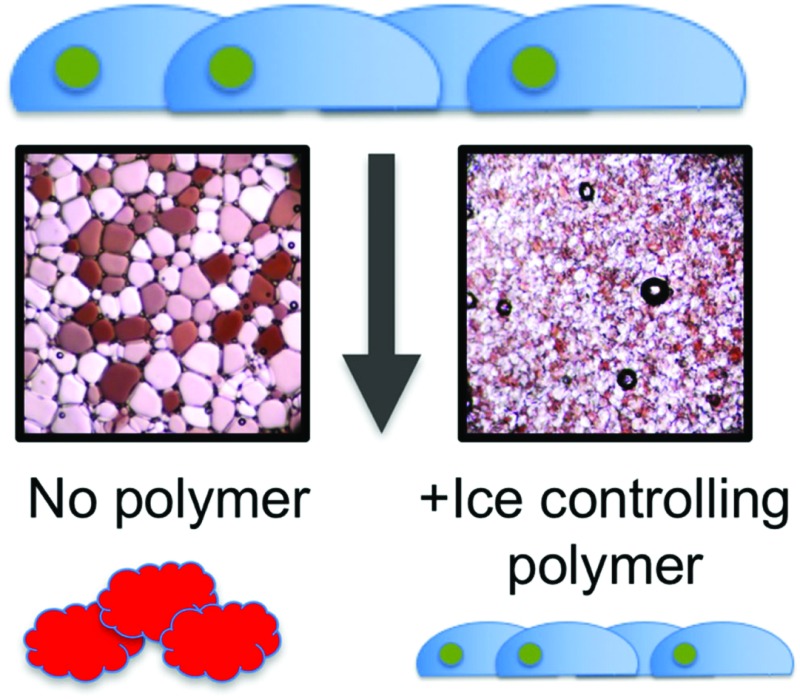
Ice growth inhibiting polymers are shown to enhance the (non-vitrifying) cryopreservation of several cell lines and also primary hepatocytes.

## Introduction

**Fig. 1:**
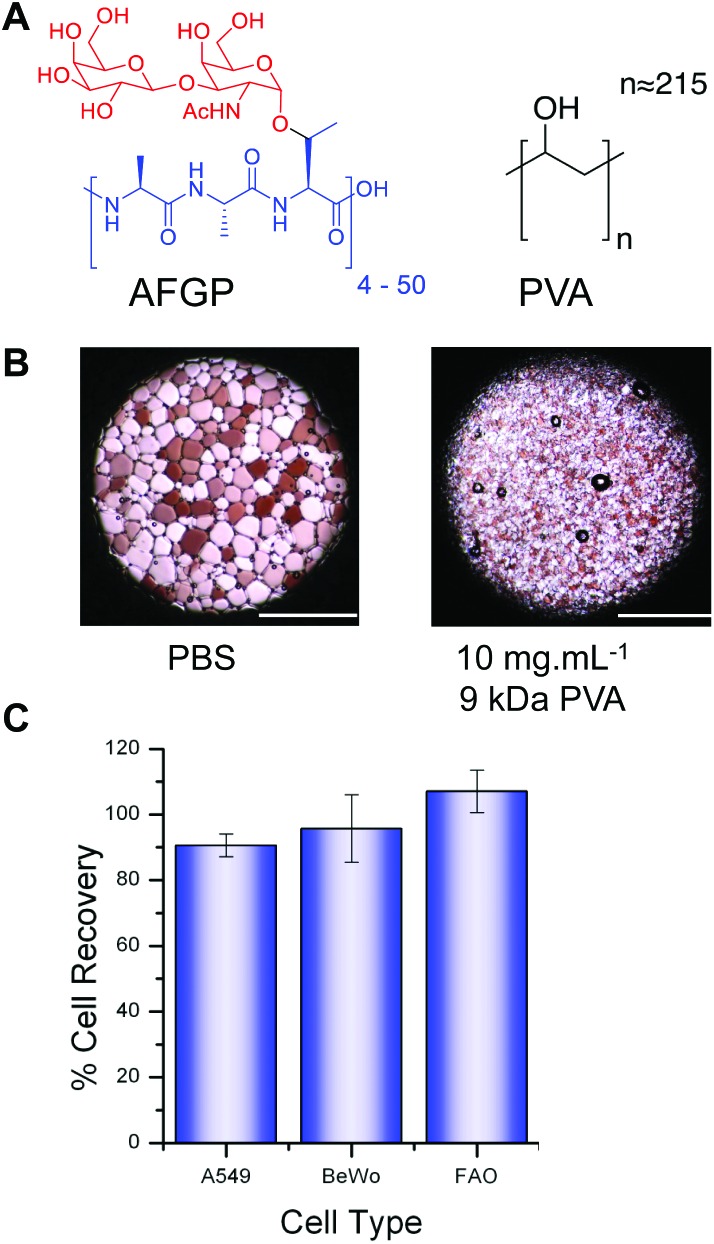
PVA structure, IRI activity and cytotoxicity. (A) Structure of AF(G)P and PVA; (B) micrographs of ice crystals annealed for 30 minutes at –6 °C showing inhibitory effect of PVA; (C) cytotoxicity testing of 25 mg mL^–1^ PVA (9 kDa) after 4 hour incubation. Cell metabolic activity measured by MTT (A549) and resazurin (BeWo and FAO) assays. Error bars represent ± standard deviation from at least 3 repeats.

The ability to store cells and tissues by cryopreservation is crucial in biochemistry, cell biology, and clinical medicine.^1,2^ Current cryopreservation strategies require the addition of large volumes (10–20 wt%) of membrane permeable organic solvents (*e.g.* glycerol or DMSO) and often precise freezing rates to promote either vitrification or dehydration and limit intracellular ice formation (nucleation) and growth.^3^ These solvents are cytotoxic in this concentration range at physiologically relevant temperatures and hence must be added/removed rapidly to reduce cellular damage, which would perturb subsequent assays/testing.^4–6^ With immortalized cells, reduced cell counts can often (but not always) be overcome by simple proliferation. This is typically not possible with primary cells *in vitro*, necessitating donors, which are a limited resource. Analysis of cryopreservation has identified that ice crystal growth (recrystallization) during the thawing process is a major cause of cell death (as is intracellular ice nucleation) and is hence a valid target for enhanced cryopreservation.^7^ The most well-known ice recrystallization inhibitors (IRIs) are antifreeze (glyco)proteins (AF(G)P)s (Fig. 1A) which can halt ice growth at sub mg mL^–1^ concentrations.^3,8–10^ Carpenter *et al.* have demonstrated that supplementing erythrocyte cryopreservation solutions with closely related antifreeze proteins (AFPs) can increase cell number post-thawing, but was limited by the onset of dynamic ice shaping (DIS). DIS was shown to incite mechanical damage that outweighed the benefits attained by IRI activity.^11^ Other attempts to employ AF(G)Ps have met with mixed results; Wang *et al.* reported that AF(G)P had a detrimental effect on rat cardiac explant cryopreservation even at concentrations as low as 10 μg mL^–1^ (ref. 12) and Koshimoto & Mazur described how AFP and AF(G)P addition had an adverse effect on spermatozoa storage.^13^ However success has been demonstrated with islet cells^14^ and oocytes.^15^ Understanding the mechanisms and optimizing the application of AF(G)Ps is also limited by their low availability from natural sources and challenging synthesis, which only yields milligrams of product.^16^ To address this, Gibson *et al.*, have demonstrated that biomimetic polymers can reproduce the most desirable properties of AF(G)Ps and are readily synthesized, highly tuneable and produced on an industrial scale.^17,18^ In particular, poly(vinyl alcohol) (PVA) has extremely potent IRI activity comparable to AF(G)Ps^19–21^ and is FDA approved for various pharmaceutical, *in vivo*, and food applications (Fig. 1A).^22^ PVA is also a potent antinucleating agent, which has been successfully used in the vitrification of organs.^23,24^ Other polymers such a poly(vinyl pyrollidone) and hydroxyethyl starch have been used as non-penetrative cryoprotectants but do not possess IRI activity.^1,17,25^ Gibson *et al.* have shown that addition of PVA (9 kDa) to red blood cells increases the yield of cells post-thawing, and even enables solvent-free storage, by inhibiting ice growth, rather than formation (nucleation).^26–28^ Whilst an interesting observation, red blood cells are anuclear and do not proliferate, meaning any toxic effects are less pronounced.

Here, we investigate the use of PVA as an ice recrystallization inhibitor to reduce cell damage during freezing of a range of nucleated, immortalized cell types (A549, BeWo and FAO) and primary rat hepatocytes, using minimal DMSO concentrations. Rapid (A549 and BeWo) and slow (FAO and Primary) freezing methods are employed and the effect of the polymer, its architecture and the cell type are studied, demonstrating this to be a simple, but effective strategy.

## Results and discussion

The chemical structure of the structurally simple, biomimetic, ice recrystallization inhibiting (IRI) polymer, PVA, is shown compared to native AF(G)P (Fig. 1A). Example ice wafers grown with and without the PVA additive are also included (Fig. 1B), demonstrating that within 30 minutes large (>200 μm) ice crystals have grown in the absence of PVA, but addition of just 1 wt% (10 mg mL^–1^) of 9 kDa PVA completely halts ice crystal growth (as we have previously reported^17^). This ability to halt recrystallization (growth) is the primary mechanism of protection to be investigated here.

Three immortalized cell types were selected, along with primary rat hepatocytes (*vide infra*) for use in this study. A549 cells (human lung adenocarcinoma) are routinely used in biochemical studies as mimics of type II alveolar epithelial cells;^29^ BeWo cells (human choriocarcinoma) are placental derived and show well-characterized responses to forskolin stimulation enabling functionality as well as viability to be evaluated;^30^ FAO cells (rat hepatoma) provide a model for primary hepatocytes with additional use in pharmacokinetic and pharmacodynamic studies.^31,32^ Cytotoxicity screening of 25 mg mL^–1^ PVA with each immortalized cell line after 4 hours of exposure (Fig. 1C) indicated there was no sign of cytotoxicity as expected for this material^22^ even at the concentration applied which is at least 10 fold higher than would be used in a cryopreservation context. PVA has been reported to undergo hydrogel formation under cryogenic conditions but at significantly higher concentrations (>10× fold) and molecular weights and was not a property observed in this system.^33^

**Fig. 2:**
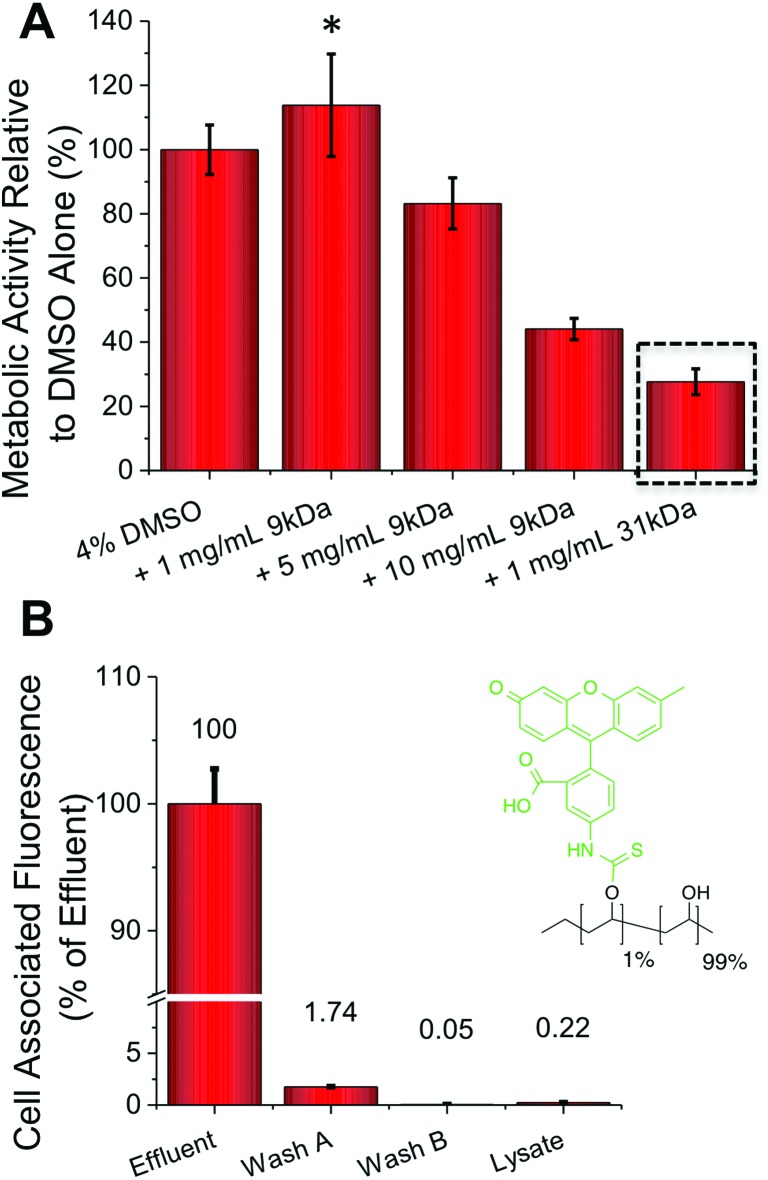
A549 Cryopreservation (rapid freezing) and FITC-PVA permeability. (A) Post freeze–thaw A549 cell metabolic activity with and without PVA additives (MTT assay); (B) FITC-labelled PVA uptake into A549 cells following 2 hours exposure at 37 °C). All data from at least 3 repeats and errors bars represent ± standard deviation. * represents *p* ≤ 0.05 relative to DMSO only.

Typical cryopreservation conditions for these cell lines would involve addition of ≈10 wt% DMSO in cell media (although many laboratories use conditions they optimise individually). To enable us to study the influence of the PVA additive, a range of trial cryopreservation tests were conducted (ESI Fig. 1†) to find the DMSO concentration, which, under our freezing conditions, yielded the greatest recovery. However it must be highlighted that the complete removal of DMSO results in zero recovery regardless of the inclusion of PVA or not. Furthermore, changes in freezing/thawing rates will obviously have an effect (*i.e.* nucleation or vitrification), but were outside of the scope of this particular study. Each cell line was cultured to confluence and then added to cryovials (1.8 mL) as 1 mL aliquots suspended in either phosphate buffered saline (PBS) or complete medium with the predetermined amounts of DMSO and PVA. Post-freezing, cells were thawed at 23 °C (air), to encourage ice recrystallization and enable the impact of IRI activity to be measured. This is also a more practical method, and representative of the storage of larger volumes (*e.g.* organs) where thermal gradients exist during thawing and rapid, homogeneous thawing is challenging. Thawed cells (A549) were plated and incubated overnight and cell metabolic activity (MTT) measured (Fig. 2).

The addition of just 1 mg mL^–1^ (0.1 wt%) of PVA to the DMSO cryopreservation solution resulted in a 20% increase in the cell (A549) metabolic activity (*i.e.* viability) post-thawing, providing a real benefit (*p*-value = 0.0331) (Fig. 2A). Interestingly, addition of higher concentrations of PVA actually lead to reduced cell viability. Cytotoxicity screening, at concentrations far greater than used in cryopreservation, indicated that this is not a toxic effect of the polymer itself, so it is instead attributed to the unwanted effect of DIS (the formation of needle-like ice crystals) at higher PVA concentrations.^20,34^ This effect was more pronounced for the higher molecular weight, 31 kDa PVA, which at just 1 mg mL^–1^ lead to a 75% decrease in metabolic activity (*p*-value <0.0001). These data highlight that the architecture and size of the polymer is crucial, as well as potent IRI activity to achieve a successful cryopreservation enhancement and a delicate balance must be struck. For example further reductions in the molecular weight of PVA tends to a complete loss of IRI activity.^17,35^ FITC (fluorescein isothiocyanate) labelled PVA was also added to confluent A549 cells (Fig. 2B). Following incubation and washing the total cell-associated fluorescence was measured, revealing close to zero PVA has been internalized or bound to the cell surface. This confirms our hypothesis that PVA is a purely extracellular-functional additive, and is particular useful for biomedical applications where all additives need to be removed post-freeze/thaw rapidly.

**Fig. 3:**
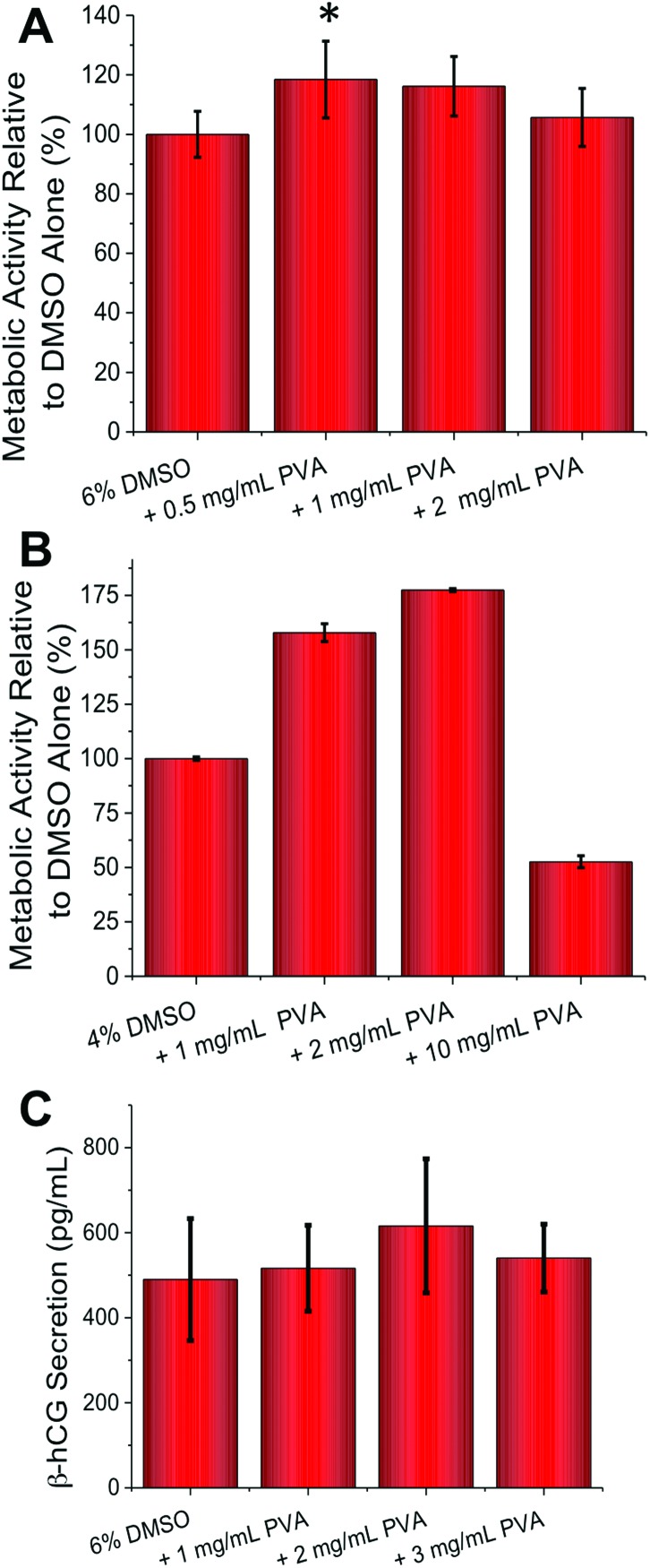
FAO (slow freezing) and BeWo (rapid freezing) cryopreservation. (A) FAO cell metabolic activity post-freeze–thaw as determined by resazurin assay; (B) BeWo cell metabolic activity post-freeze–thaw as determined by resazurin assay; (C) β-hCG secretion (pg mL^–1^) due to forskolin induced BeWo cell syncytialization, post cryopreservation. All data from at least 3 repeats and errors bars represent ± standard deviation. * represents *p* ≤ 0.05 relative to DMSO only.

Guided by the above results, cell-freezing using the FAO and BeWo cell lines were conducted. With FAO cells (Fig. 3A) peak enhancement was observed with 0.5 mg mL^–1^ of PVA (*p*-value = 0.0129), contrasted to 1 mg mL^–1^ for A549 which suggests optimum conditions for each cell line need to be determined, based on their own responses to cold-shock. As with A549 an increase in viability of 20% was observed, which is significant considering the minute quantities of PVA being applied. With BeWo cells (Fig. 3B) the observed increase in metabolic activity post-thaw was a remarkable 75% with 2 mg mL^–1^ PVA (9 kDa) (*p*-value <0.0001). This obviously huge increase demonstrates that under the correct conditions recrystallization inhibitors can dramatically improve cryopreservation. As seen with FAO cells, higher concentrations or addition of 31 kDa PVA (data not shown) lead to reductions in cell viability. These experiments clearly show that IRI active polymers are a useful tool in cell cryopreservation and can significantly enhance routine cell preservation without disruption to standard protocols, but that the optimized concentration required for each cell type varies.

The above assays only probed cellular metabolic activity, but we also wanted to probe if cellular function was affected by addition of the PVA. BeWo cells syncytialize and secrete β-hCG (human pregnancy hormone) when exposed to forskolin (50 μM) necessitating significant changes in gene expression. A quantitative ELISA assay (Fig. 3C) revealed that β-hCG levels were identical (or slightly higher, but not statistically significant) in the PVA cryopreserved cells compared to DMSO only, meaning that complex cellular function was unaffected by this freezing method. It should be noted that β-hCG secretion does not correlate linearly with cell number as cell-to-cell contact (density) influences the rate and extent of syncytialization.

**Fig. 4:**
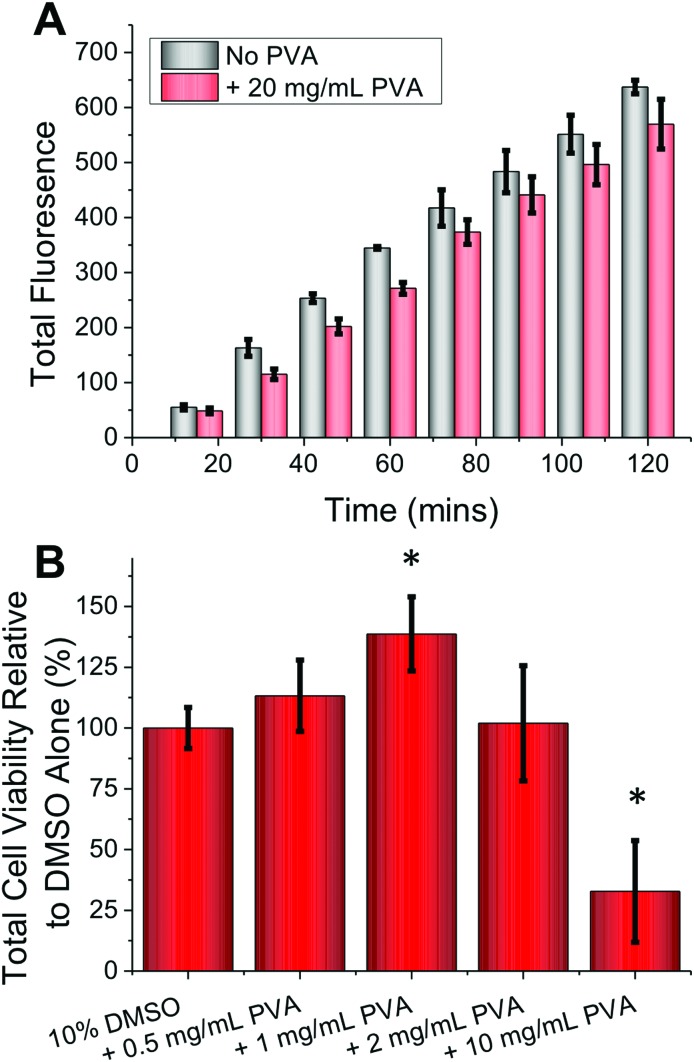
Rat hepatocyte cytotoxicity and cryopreservation (slow freezing). (A) Resazurin fluorescence (cell metabolic activity assay) over 2 hours in rat hepatocytes with and without of 20 mg mL^–1^ PVA; (B) cell metabolic activity (%) of cryopreserved rat hepatocytes (1 × 10^6^ cells per mL) each plated overnight, relative to 10% (v/v) DMSO preservation alone as judged by the resazurin assay after 6 hours. Freezing in complete medium alone gave 0% cell recovery; all data from at least 3 repeats and errors bars represent ± standard deviation. * represents *p* ≤ 0.05 relative to DMSO only.

As a final test of this technique, primary cells were employed as these are more sensitive and challenging to cryopreserve and are less straightforward to culture, hence maximizing their recovery is essential.^1,36^ Primary cells also tend to be less tolerant to environmental perturbations *ex vivo*. Guided by the positive results with the FAO cells, primary rat hepatocytes were harvested (sprague dawley) and prepared according standard (percoll) protocols.^37^ No cytotoxicity was observed at concentrations as high as 20 mg mL^–1^ PVA (Fig. 4A) (*p*-value = 0.0673 (120 minutes)), nor were there any morphological changes. These cells were cryopreserved using a slow-freeze methodology, then post cryopreservation, metabolic activity was measured (resazurin) after plating and incubating cells overnight. Compared to DMSO alone, addition of 1 mg mL^–1^ PVA gave >30% increase in cell recovery (Fig. 4B) (*p*-value <0.001). As observed with the immortalized cells, higher concentrations of PVA (10 mg mL^–1^) led to reduced metabolic activity (*p*-value <0.001) used as an indicator of cell recovery associated with DIS, which is mitigated by limiting the concentration of IRI agent applied. These results effectively demonstrate that IRI active compounds can indeed enhance cellular cryopreservation, without requiring significant changes to existing protocols making it a broadly applicable method across a range of disciplines that employ cell culture.

## Conclusions

Here the use of PVA to modulate ice crystal growth (IRI) during cell freeze/thawing is described. Addition of just 1–3 mg mL^–1^ of PVA (9 kDa) to standard DMSO freezing protocols resulted in an increase of 20–75% in total cell metabolic activity relative to DMSO alone. This remarkable increase is being achieved despite the weight fraction of the polymer additive being only 0.1–0.3 wt%. The mechanism of protection is linked to ice growth inhibition, rather than nucleation inhibition (which is crucial in vitrification). Functional assays on BeWo cells demonstrated functionality retention by measurement of β-hCG secretion, in addition to metabolic activity assays. Finally, freshly isolated primary rat hepatocytes cells were also stored successfully using this approach with PVA giving a ≈35% increase in cell metabolic activity relative to DMSO alone. This is particularly important in the context of cryopreservation and distribution of human tissue for transplantation. The mode of action of PVA was shown to be purely extracellular, with no polymer permeating inside the cell, enabling its rapid removal post-thawing. As PVA is commercially available to GMP standards, and already has broad use in the pharmaceutical sciences and food industry, it may find wide-ranging applicability in cryopreservation scenarios.

## Experimental section

### Materials

All chemical and biochemical reagents were purchased from Sigma-Aldrich Ltd (UK) or Life Technologies Ltd (UK) and used without further purification except PVA. 9 kDa PVA (average *M*_w_ 9000–10 000, 80% hydrolysed) and 31 kDa PVA (average *M*_w_ 31 000–50 000, 98–99% hydrolysed) were purified further by dialysis (4 L H_2_O with at least 5 changes) against 1000 MWCO membranes (Spectrum Laboratories Inc., CA, USA) to remove any small molecule weight contaminants, prior to freeze drying to yield a lyophilized solid for further use. Each immortalised cell line used was derived from stocks originally obtained from the American Type Culture Collection (ATCC) (VA, USA) and propagated using standard cell culture practices prior to use.

### Ice recrystallization inhibition assay

Ice recrystallization inhibition (IRI) was investigated using a modified “splat” assay according to previously published protocols.^10,25^ In brief, a 10 μL droplet is expelled onto a no. 0 thickness coverslip placed upon a pre-cooled (CO_2(s)_) aluminium (below a minimum of –40 °C) plate creating a polycrystalline wafer containing ice crystals (<10 μm in diameter). The coverslip is immediately transferred to a N_2(l)_ cooled linkam BCS196 cryostage (Linkam Scientific Instruments Ltd, UK.) at –6 °C for 30 minutes prior to imaging using an Olympus CX41 microscope equipped with a UIS-2 10×/0.25/N/–/FN22 lens, cross polarizers (Olympus Ltd, Southend on sea, UK) and a Canon EOS 500D SLR digital camera (Canon (UK) Ltd, Surrey, UK). The sizes of the ten largest crystals were measured using the freely available image-processing software ImageJ^38^ and the mean largest grain size expressed as a percentage of a PBS control.

### Cytotoxicity assessment of PVA to A549, BeWo, FAO and primary rat hepatocytes

Purified PVA (9 kDa) was dissolved in PBS at 25 mg mL^–1^ and incubated with A549, BeWo and FAO cells for 4 hours in a 37 °C/5% CO_2(g)_ humidified incubator. Immediately afterwards cells were washed with PBS and cell viability measured by standard MTT^39^(A549) and resazurin^40^ (BeWo and FAO) assays. Metabolic activity was expressed as a % against untreated controls (in the absence of DMSO) with the mean values of at least 3 replicates reported. Primary rat hepatocytes (8 × 10^5^ cells) were plated in triplicate onto 60 mm *∅* collagen (type I) coated plates (BD Biosciences, CA, USA) for 3 hours in complete (DMEM) medium at 37 °C. Afterwards cells were washed twice with PBS to remove any non-adherent cells and debris and then replaced with complete medium for 36 hours at which point the inherent metabolic activity was assessed by the non-destructive resazurin assay (enabling measurements at multiple time points). Afterwards, these primary rat hepatocytes were treated with 20 mg mL^–1^ PVA in complete medium for 4 hours, washed and then viability assessed by the resazurin assay. The total fluorescence before and after PVA addition was taken at 20 minute intervals for a period of 2 hours allowing the effect of PVA to be investigated with the mean values of at least 3 replicates reported.

### Cryopreservation of A549 and BeWo cells

A549 and BeWo cells were subjected to a rapid freezing protocol. Confluent cells (RPMI containing 10% (v/v) FBS plus 100 U per mL Penicillin, 100 μg per mL streptomycin) were trypsinised, washed and resuspended in PBS containing the appropriate amount of DMSO and desired PVA (in PBS) to a volume of 1 mL in 1.8 mL cryovials (between 1 × 10^5^–1 × 10^6^ cells per mL). Cells without PVA were given an equivalent volume of PBS and DMSO. Preparations were mixed by inversion to ensure homogeneity prior to immersion in a (–78 °C) IPA/CO_2(s)_ slurry for 60 seconds and were enclosed in CO_2(s)_ for a period of 20 minutes. Cells were thawed slowly at 23 °C in air to maximize potential ice recrystallization. Once thawed, cells were plated as a monolayer in complete medium in 48/96 well plates and incubated overnight in a 37 °C/5% CO_2(g)_ humidified incubator prior to viability and functionality assays.

### Syncytialization and functionality assessment of BeWo cells

After cryopreservation BeWo cells were plated as a monolayer in a 48-well plate overnight to enable adhesion prior to the addition of 50 μM forskolin (10 mM stock in DMSO) in complete medium for 72 hours enabling syncytialization. The production of β-HCG was investigated using a β-HCG ELISA kit specific for detecting β-HCG present within cell culture medium with the mean values of at least 3 replicates reported.

### Cryopreservation of FAO cells and primary rat hepatocytes

FAO and primary rat hepatocytes were subjected to a slow freezing protocol. Confluent or freshly isolated cells were trypsinised (as appropriate), washed and resuspended in medium (RPMI for FAO and DMEM for primary hepatocytes) containing 10% (v/v) FBS plus 100 U per mL penicillin, 100 μg per mL streptomycin with the appropriate amount of DMSO and desired PVA (in complete medium) to a volume of 1 mL in 1.8 mL cryovials. FAO cells were cryopreserved at a concentration between 1 × 10^5^–1 × 10^6^ cells per mL and primary rat hepatocytes at 1 × 10^6^ cells per mL. Cells without PVA were given an equivalent volume of medium. Cells were then cooled to 4 °C in a Nalgene “Mr. Frosty™” containing IPA over a period of 120 minutes. Cooled cells were transferred to –80 °C for between 24–72 hours and finally placed into vapour phase N_2(l)_ (–196 °C) overnight. Cells were thawed slowly at 23 °C in air to maximize potential ice recrystallization. Once thawed, FAO cells were plated as a monolayer in complete medium in 48/96 well plates and incubated overnight in a 37 °C/5% CO_2(g)_ humidified incubator prior to viability and functionality assays. Thawed primary rat hepatocytes were plated as a monolayer on 60 mm *∅* collagen (type I) coated plates and incubated for 3 hours. Primary rat hepatocytes were then washed twice with PBS to remove any non-adherent cells and debris and then replaced with complete medium and incubated overnight in a 37 °C/5% CO_2(g)_ humidified incubator prior to viability and functionality assays.

### Data analysis

All statistics and calculations for (%) cell recovery and (%) mean largest grain size were determined using Microsoft Excel 2008 for Mac. Significance determination for cryopreservation data used a two-tailed homoscedastic Student's *t*-test with a 95% confidence interval (*P*-value ≤0.05) comparing the value of stated interest with the relevant control.

## References

[cit1] Meryman H. T. (2007). Transfusion.

[cit2] Fowler A., Toner M. (2005). Ann. N. Y. Acad. Sci..

[cit3] Mazur P. (1984). Am. J. Physiol..

[cit4] Tjernberg A., Markova N., Griffiths W. J., Hallén D. (2006). J. Biomol. Screening.

[cit5] Trubiani O., Salvolini E., Staffolani R., Di Primio R., Mazzanti L. (2003). Int. J. Immunopathol. Pharmacol..

[cit6] Sperling S., Larsen I. G. (2009). Acta Ophthalmol..

[cit7] Karlsson J. O., Cravalho E. G., Borel Rinkes I. H., Tompkins R. G., Yarmush M. L., Toner M. (1993). Biophys. J..

[cit8] Harding M. M., Anderberg P. I., Haymet A. D. J. (2003). Eur. J. Biochem..

[cit9] Ewart K. V., Lin Q., Hew C. L. (1999). Cell. Mol. Life Sci..

[cit10] Knight C. A., Hallett J., Devries A. L. (1988). Cryobiology.

[cit11] Carpenter J. F., Hansen T. N. (1992). Proc. Natl. Acad. Sci. U. S. A..

[cit12] Wang T., Zhu Q., Yang X., Layne Jr. J. R., Devries A. L. (1994). Cryobiology.

[cit13] Koshimoto C., Mazur P. (2002). Cryobiology.

[cit14] Matsumoto S., Matsusita M., Morita T., Kamachi H., Tsukiyama S., Furukawa Y., Koshida S., Tachibana Y., Nishimura S.-I. I., Todo S. (2006). Cryobiology.

[cit15] Jo J. W., Jee B. C., Suh C. S., Kim S. H. (2012). PLoS One.

[cit16] Wilkinson B. L., Stone R. S., Capicciotti C. J., Thaysen-Andersen M., Matthews J. M., Packer N. H., Ben R. N., Payne R. J. (2012). Angew. Chem., Int. Ed..

[cit17] Congdon T., Notman R., Gibson M. I. (2013). Biomacromolecules.

[cit18] Phillips D., Congdon T., Gibson M. I. (2016). Polym. Chem..

[cit19] Gibson M. I., Barker C. A., Spain S. G., Albertin L., Cameron N. R. (2009). Biomacromolecules.

[cit20] Budke C., Koop T. (2006). ChemPhysChem.

[cit21] Inada T., Lu S.-S. (2003). Cryst. Growth Des..

[cit22] DeMerlis C., Schoneker D. (2003). Food Chem. Toxicol..

[cit23] Wang H.-Y., Inada T., Funakoshi K., Lu S.-S. (2009). Cryobiology.

[cit24] Fahy G. M., Wowk B., Wu J., Phan J., Rasch C. M., Chang A., Zendejas E. (2004). Cryobiology.

[cit25] Deller R., Congdon T., Sahid M. A., Morgan M., Vatish M., Mitchell D. A., Notman R., Gibson M. I. (2013). Biomater. Sci..

[cit26] Deller R., Vatish M., Mitchell D. A., Gibson M. I. (2014). Nat. Commun..

[cit27] Deller R., Mitchell D. A., Vatish M., Gibson M. I. (2015). ACS Biomater. Sci. Eng..

[cit28] Mitchell D. E., Lovett J. R., Armes S. P., Gibson M. I. (2016). Angew. Chem., Int. Ed..

[cit29] Lieber M., Todaro G., Smith B., Szakal A., Nelson-Rees W. (1976). Int. J. Cancer.

[cit30] Orendi K., Gauster M., Moser G., Meiri H., Huppertz B. (2010). Reproduction.

[cit31] Reuber M. D. (1961). J. Natl. Cancer Inst..

[cit32] Donato M. T., Gomezlechon M. J., Castell J. V. (1993). Anal. Biochem..

[cit33] Hassan C. M., Peppas N. A. (2000). Macromolecules.

[cit34] Inada T., Lu S.-S. (2004). Chem. Phys. Lett..

[cit35] Budke C., Dreyer A., Jaeger J., Gimpel K., Berkemeier T., Bonin A. S., Nagel L., Plattner C., Devries A. L., Sewald N., Koop T. (2014). Cryst. Growth Des..

[cit36] Sosef M. N., Baust J. M., Sugimachi K., Fowler A., Tompkins R., Toner M. (2005). Ann. Surg..

[cit37] Neufeld D. S., Isolation Rat Liver Hepatocytes, Basic Cell Culture Protocols, Totowa, NJ, 2nd edn, 1997.

[cit38] Abràmoff M., Magalhaes P., Ram S. J. (2004). Biophotonics Int..

[cit39] Mosmann T. (1983). J. Immunol. Methods.

[cit40] O'Brien J., Wilson I., Orton T., Pognan F. (2000). Eur. J. Biochem..

