# Supplementary material for: Enhanced non-vitreous cryopreservation of immortalized and primary cells by ice-growth inhibiting polymers
Source: Biomater Sci. 2016 May 6;4(7):1079–84. doi: 10.1039/c6bm00129g (PMC4918798; doi:10.1039/c6bm00129g)
Supplement: Supplementary file 1 [file BM-004-C6BM00129G-s001.pdf]

Enhanced Non-Vitreous Cryopreservation of Immortalized and Primary Cells By Ice-Growth Inhibiting Polymers – *Supplementary Information.*

Robert. C. Deller,[ab†] Jeffrey E. Pessin,[cd] Manu Vatish,[e] Daniel A.Mitchell,[f] and Matthew I.Gibson.[a,f*]

[a]Department of Chemistry, University of Warwick, CV4 7AL, UK.
[b]Molecular Organisation & Assembly of Cells DTC, University of Warwick, CV4 7AL, UK.
[c]Department of Medicine, Albert Einstein College of Medicine, Bronx, New York, USA.
[d]Department of Molecular Pharmacology, Albert Einstein College of Medicine, Bronx, New York, USA.
[e]Nuffield Department of Obstetrics & Gynaecology, University of Oxford, OX3 9DU, UK.
[f]Warwick Medical School, University of Warwick, CV4 7AL, UK.
[*]Denotes corresponding author.
[†]Current address: School of Cellular & Molecular Medicine, University of Bristol, Bristol, BS8 1TD, UK.

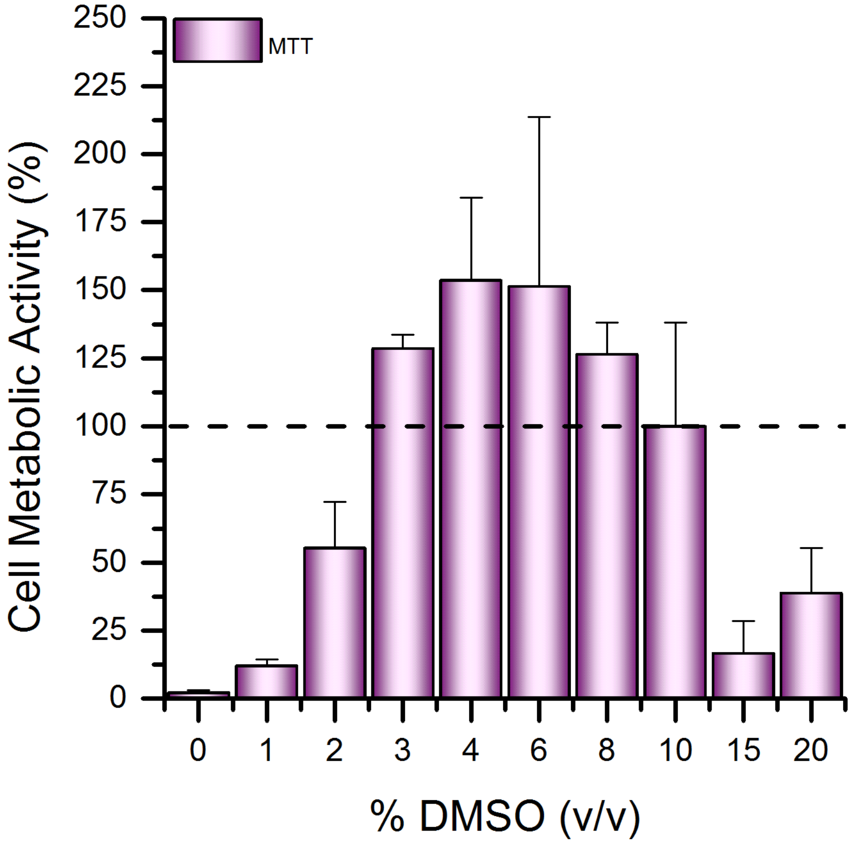

Supplementary Figure 1. Optimizing DMSO concentration during cryopreservation. Cell viability (MTT) of A549 cells post cryopreservation with various DMSO concentrations. All data from at least 3 repeats and errors bars represent ± standard deviation.
